# Evaluation of the Disinfection Efficacy of Er: YAG Laser Light on Single-Species Candida Biofilms—An In Vitro Study

**DOI:** 10.3390/dj13020088

**Published:** 2025-02-19

**Authors:** Diana Dembicka-Mączka, Małgorzata Kępa, Jakub Fiegler-Rudol, Zuzanna Grzech-Leśniak, Jacek Matys, Kinga Grzech-Leśniak, Rafał Wiench

**Affiliations:** 1 EMDOLA Student, Department of Periodontal and Oral Mucosa Diseases, Wroclaw Medical University, 50-425 Wroclaw, Poland; dianadembicka@vp.pl; 2 Department of Microbiology, Faculty of Pharmaceutical Sciences in Sosnowiec, Silesian Medical University, 41-902 Katowice, Poland; mkepa@sum.edu.pl; 3 Department of Periodontal and Oral Mucosa Diseases, Faculty of Medical Sciences in Zabrze, Medical University of Silesia, 40-055 Katowice, Poland; s88998@365.sum.edu.pl; 4 Student Faculty of Dentistry, Wroclaw Medical University, 50-425 Wroclaw, Poland; zuzanna.grzech-lesniak@student.umw.edu.pl; 5 Dental Surgery Department, Wroclaw Medical University, 50-425 Wroclaw, Poland; jacek.matys@umw.edu.pl; 6 Department of Periodontics, School of Dentistry, Virginia Commonwealth University, Richmond, VA 23284, USA

**Keywords:** oral candidiasis, denture stomatitis, 2940 nm, ablation

## Abstract

**Background/Objectives:** Oral candidiasis is an opportunistic infection caused by Candida species. Recently, antifungal drugs have become less effective due to yeast resistance, emphasizing the need for new treatment strategies. This study aimed to assess the effect of the Er:YAG laser on the inhibition of growth and elimination of mature single-species Candida biofilms. **Methods:** The study utilized reference strains of *C. albicans*, *C. glabrata*, *C. parapsilosis*, and *C. krusei* organized in single-species biofilms on Sabouraud dextrose agar (SDA). First part: *Candida* suspensions (0.5 McFarland standard) were spread on SDA plates—two for each strain. Er:YAG laser irradiation was applied in a single pulse mode, 30 to 400 mJ, to 32 predetermined points. The growth inhibition zones (GIZs) were measured at 24–96 h of incubation. Second part: biofilms were prepared similarly and, after 96 h of incubation, exposed to Er:YAG laser irradiation at different energies (50, 100, 150, 200 mJ) for 180 s, per 1.44 cm area. Post-irradiation, impressions were taken using Rodac Agar to determine yeast counts. The count of colony-forming units (CFU) after irradiation was measured and results were analysed statistically. **Results**: First part: GIZ was found in all irradiated sites, with various *Candida* strains. The results showed a significant increase in the width of GIZ in the energy range of 30–280 mJ and a non-significant increase in the energy range of 300–400 mJ. Second part: the number of CFU remaining after the irradiation of biofilms with 150 mJ energy differed statistically significantly from other results obtained after using 50, 100, or 200 mJ energy, regardless of the *Candida* strain tested. **Conclusions**: The Er:YAG is shown to have good disinfecting properties (inhibiting biofilm growth, even at low-energy doses (50 mJ), and eliminating maturity, *Candida* spp. biofilms most effective on the 150 mJ energy dose).

## 1. Introduction

### 1.1. Background

Oral candidiasis is an opportunistic infection primarily caused by *Candida* species [1]. Among them, *C. albicans* remains the most frequently isolated pathogen (47–84% of oral yeast infections) [2,3,4]. However, long-term epidemiological studies have reported a rising prevalence of non-albicans *Candida* (NAC) species, including *C. dubliniensis*, *C. glabrata*, *C. krusei*, *C. kefyr*, *C. parapsilosis*, *C. stellatoidea*, and *C. tropicalis* [5,6,7,8,9,10]. This shift is attributed to excessive antifungal drug exposure, advancements in diagnostic techniques, climate change, and recent global pandemics [11,12,13]. Additionally, *C. auris*, a multidrug-resistant species, has emerged as a significant clinical challenge, causing severe infections with mortality rates reaching 72%, particularly in immunocompromised individuals [14,15]. The development of oral candidiasis is influenced by a range of local and systemic predisposing factors that disrupt the host–microbial balance. Local risk factors include wearing dental prosthetics [16,17,18,19,20,21,22], reduced salivary flow, poor oral hygiene, mucosal trauma [23,24], and the use of local antibiotics or corticosteroids [25,26]. Systemic predisposing conditions encompass immunosuppressive diseases (e.g., HIV/AIDS, malignancies, chemotherapy) [27], endocrine disorders (e.g., diabetes mellitus) [27], nutritional deficiencies (e.g., iron, folate, vitamin B12) [28,29], broad-spectrum antibiotic use [30], extremes of age (neonates and elderly) [31], and hormonal changes (e.g., pregnancy) [32]. Beyond its role in mucosal infections, *Candida* spp. may contribute to the carcinogenesis of oral epithelium, increasing the risk of squamous cell carcinoma through nitrosamine and acetaldehyde production [33,34]. Additionally, candidemia represents a major hospital-acquired bloodstream infection (BSI) with mortality rates reaching 50% [35,36,37,38,39]. Due to their ability to form multi-species biofilms with bacteria such as *Streptococcus gordonii*, *S. salivarius*, and *S. oralis*, NAC species exhibit enhanced resistance to antifungals, attributed to cell wall modifications such as reduced ergosterol content and altered β-glucan structure [4,40]. Therapeutic challenges, including suboptimal antifungal dosing and premature treatment discontinuation, have led to recurrent infections and prolonged therapy requirements [41]. Given these challenges, alternative therapeutic strategies are needed to supplement or replace conventional antifungal pharmacotherapy. Promising approaches include chlorhexidine, cetylpyridinium, sanguinarine, octenidine, heavy metal nanoparticles, ozone, and laser therapy [42,43,44,45,46,47,48,49]. Among these, laser therapy has gained attention, with techniques such as antimicrobial photodynamic therapy (aPDT) utilizing photosensitizers (e.g., toluidine blue ortho, methylene blue, curcumin) [50,51]. Additionally, high-power lasers—including surgical diode lasers, Nd:YAG, Er:YAG, and Er,Cr:YSGG—have demonstrated antimicrobial efficacy [52,53,54,55,56,57,58].

The Er:YAG laser appears to be a promising tool for biofilm elimination due to its high affinity for water [54,55,56,57]. The 2940 nm wavelength of Er:YAG laser light is near the peak absorption of water, making it highly effective in targeting microbial cytoplasmic water content [42,53]. However, research on Er:YAG laser effectiveness against Candida biofilm remains limited, with few studies investigating its direct antifungal efficacy. This study aimed to bridge this gap by evaluating the potential of Er:YAG laser irradiation in inhibiting *Candida* biofilm growth and eliminating mature biofilms [42,43,44,45,46,47,48,49,50,51,52,53,54].

The laser usage protocol in this study involved the use of an Er:YAG laser (AdverEvo, Morita, Osaka, Japan) for *Candida* biofilm disinfection through two experimental approaches: (1) growth inhibition, where *Candida* suspensions (0.5 McFarland standard) were spread on Sabouraud dextrose agar (SDA) plates and irradiated in single-pulse mode (1 Hz, 300 µs pulse duration) with varying pulse energy (30–400 mJ), assessing growth inhibition zones over 96 h, and (2) biofilm elimination, where preformed *Candida* biofilms (96 h incubation) were irradiated at four energy settings (50–200 mJ) using a 10 Hz frequency and 300 µs pulse duration, with biofilm viability evaluated through Rodac Agar impressions and CFU counts post-irradiation.

Compared to other laser types, the Er:YAG laser enables precise tissue ablation with minimal thermal damage, thereby reducing collateral tissue effects. Although extensively used in endodontics, periodontology, and peri-implantitis management for microbial decontamination [55,56,57,58], limited research explored its direct antifungal effects, particularly against *Candida* biofilms. Previous studies suggested that erbium-family lasers may effectively treat erosive lichen planus, especially in cases of *Candida* co-infection or superficial infections like black hairy tongue [59,60,61]. Given the persistent challenge of antifungal resistance in *Candida* biofilms, the application of Er:YAG laser therapy warrants further investigation.

Accordingly, this study aimed to assess the disinfecting effect of the Er:YAG laser on the inhibition of growth and elimination of mature single-species *Candida* biofilms. This study builds upon prior research on antimicrobial properties of Er:YAG lasers, including applications in endodontics, periodontology, and implant surface decontamination [62,63,64,65,66,67,68]. By applying Er:YAG laser irradiation to *Candida* biofilms, we aimed to expand its known antimicrobial capabilities to fungal infections of the oral mucosa, particularly those resistant to conventional antifungal therapies.

### 1.2. Study Hypothesis

We hypothesized that Er:YAG laser irradiation exhibits significant antifungal activity against *Candida* biofilms, demonstrating both growth inhibition and biofilm elimination. We further anticipated that its efficacy would be dose-dependent, with the highest antifungal effects observed at specific energy thresholds. This study aimed to expand the known antimicrobial applications of Er:YAG lasers, evaluating their potential role in managing oral candidiasis, particularly in cases resistant to conventional antifungal therapies.

## 2. Materials and Methods

### 2.1. Study Design

**Figure 1:**
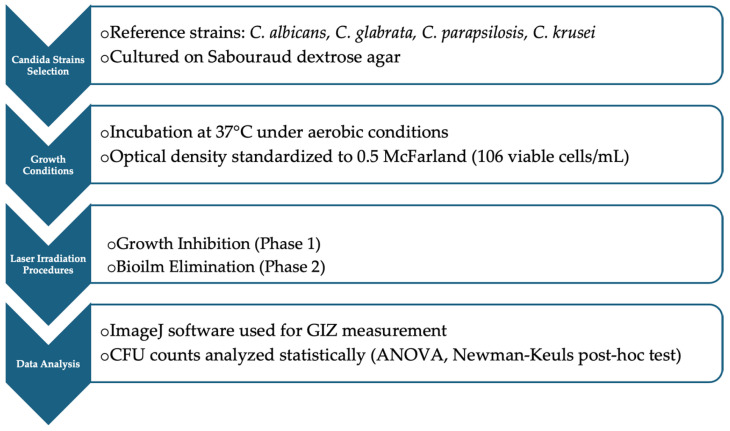
Methodological workflow of Er:YAG laser disinfection study on *Candida* biofilms.

This in vitro study evaluated the disinfection efficacy of Er:YAG laser light on single-species Candida biofilms. The study was conducted in two phases: (1) growth inhibition assessment and (2) biofilm elimination testing. The independent variable was the Er:YAG laser energy level, while the dependent variables included the growth inhibition zone and the reduction in colony-forming units. The experiment was performed in triplicate for each strain and laser setting. Sample size determination was based on preliminary trials to ensure statistically significant differences in inhibition zones and biofilm elimination. A summary of the methodological workflow is shown in Figure 1.

### 2.2. Null Hypothesis

The null hypothesis (H_0_) for the study was that Er:YAG laser irradiation would have no significant antifungal effect on Candida biofilms, meaning it would not significantly inhibit biofilm growth or reduce the viability of mature biofilms, regardless of the energy settings used.

### 2.3. Organisms

The research was carried out on reference strains of *Candida* from the American Type Culture Collection (ATCC, Manassas, VA, USA): *C. albicans* ATCC 10231, *C. glabrata* ATCC 90030, *C. parapsilosis* ATCC 22019, and *C. krusei* ATCC 6258. The yeasts were chosen for their prevalence as etiological agents of oral candidiasis [1]. These strains were part of the strain banked at the Department of Microbiology, Faculty of Pharmaceutical Sciences in Sosnowiec, Medical University of Silesia in Katowice. They were stored at −80 °C, in tryptose-soy broth with the addition of glycerol. The species chosen (*C. albicans*, *C. glabrata*, *C. parapsilosis*, and *C. krusei*) are among the most clinically relevant due to their frequent involvement in oral infections. The number of probes used in this study was determined based on previous studies evaluating the antimicrobial effects of Er:YAG laser irradiation, ensuring statistical reliability and reproducibility of the results [52].

### 2.4. Growth Conditions

To multiply and check the purity, cultures of each strain separately were placed onto Sabouraud dextrose agar (SDA) with chloramphenicol (bioMerieux, Marcy l`Etoile, France) and incubated in atmospheric air at 37 °C. After 24 h of incubation, a sample of colonies was removed from the surface of the agar plate and suspended in saline solution (0.9% NaCl). The number of viable cells in suspension was counted in a spectrophotometer Densi-La-Meter II (Erba Lachema, Brno, Czech Republic) at a wavelength of 525 nm and using the optical density of McFarland standard number 0.5 equivalent to 10^6^ viable cells/mL.

### 2.5. Study Groups

One hundred mL aliquots of a fresh suspension of each analysed *Candida* strain were colonized on the entire surface of a solid Sabouraud dextrose agar plate on a Petri dish. Eight such agar plates were prepared, with two for each test strain. A biofilm grown on the Petri dish’s surface not irradiated with Er:YAG laser was used as a control group.

### 2.6. Evaluation of the Efficacy of the Er:YAG Laser in Inhibiting the Growth of Single-Species Candida Biofilms

A previously prepared template was placed under the Petri dish with SDA and appropriate *Candida* suspension. There were 32 points, and they were arranged in the form of two concentric circles spaced 20 mm apart. Points indicated where Er:YAG laser, AdverEvo (Morita, Osaka, Japan), irradiation was performed (6 h after preparing the Petri dishes). The laser settings: single-pulse mode (frequency 1 Hz), pulse duration time 300 µs, laser tip PS600T (diameter 0.06 cm, an area of approximately 0.0028 cm^2^), no water, no air, pulse energy (30–400 mJ), fluence (0.0032–0.043 J/cm^2^), pulse power (0.3–4.0 W), power density (35,714.28–476,190.42 W/cm^2^). The laser handpiece was attached to the stand during the irradiation, so the laser tip end was at a constant distance of 10 mm from the plate surface (Figure 1).

Four repetitions (*n* = 4) were performed for each energy/power and each used *Candida* strain. After irradiation, the plates were incubated aerobically at 37 °C for 48 h. After incubation, images of the plates were acquired using a D70s Nikon camera (Nikon Corporation, Tokyo, Japan) equipped with a 6-megapixel APS-C DSLR with a 1/8000 s max shutter speed, ISO 200–1600, 3 fps burst, a 2-inch LCD, and paired with a Nikon DX AF-S NIKKOR 18–70 mm f/3.5–4.5 lens. These images were taken under white light illumination and using fixed camera settings from a standardized distance of 34 cm. The growth of the yeast colonies on irradiated agar plates was examined after aerobic growth at 37 °C after 24, 48, 72, and 96 h.

ImageJ-Fiji version 1.53j (US National Institutes of Health, Bethesda, MD, USA) software platform was used for calculating the diameter of the growth inhibition zone (GIZ) after 24 h of incubation at all 5 scheduled observation times. The results are given in millimetres (mm). The measurement was carried out after calibrating the size (measuring cup), from the left to the right border of the zone of inhibition of the growth of microorganisms parallel to the base of the photo so that the line passed through the centre of the GIZ. The results were saved in Microsoft Office Excel (Microsoft, WA, USA) and subjected to statistical evaluation.

### 2.7. Evaluation of the Efficacy of the Er:YAG Laser in Eliminating Mature Single-Species Candida Biofilms

**Figure 2:**
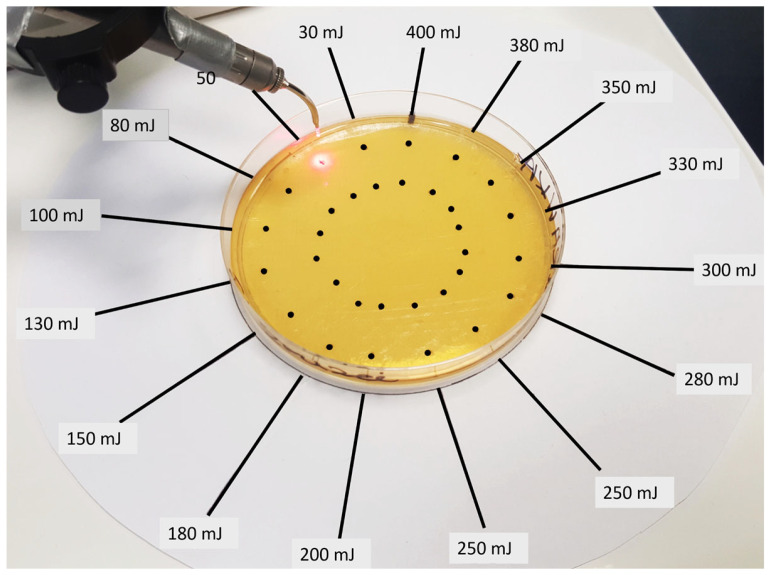
The test rig consisted of a Petri dish containing Sabouraud dextrose agar, with 100 µL of *Candida* suspension placed on a template marked with 32 dots. These dots indicate the locations for Er:YAG laser irradiation (AdverEvo, Morita, Osaka, Japan). The laser tip was positioned at a constant distance of 10 mm from the surface of the dish.

In this part of the experiment, a previously prepared template (Figure 2 was placed under the Petri dish. There were 4 squares, with sides 12 mm long. The squares indicated the places where Er:YAG laser AdverEvo (Morita, Osaka, Japan) irradiation was performed 96 h after aerobic incubation of Petri dishes at 37 °C for 48 h (the method of biofilm preparation was the same as in the first part of the experiment) (Figure 2).

The laser setting: pulse mode (frequency 10 Hz), pulse duration time 300 µs, laser tip PS600T (diameter 0.06 cm, an area of approximately 0.0028 cm^2^), angle of the laser tip end to the surface about 45°, contact mode, irradiation time 180 s/square, no water, no air, no aiming, pulse energy (50, 100, 150, 200 mJ), pulse power fluence (17.85; 35.71; 53.57; 71.42 J/cm^2^, pulse power (0.5, 1.0, 1.5, 2.0 W), pulse power density 59,523.8, 11,9047.6, 17,8571.4, 238,095.23 W/cm^2^. The selected energy parameters resulted from the analysis of the results of the first part of the experiment, where low energies suggested high disinfection efficiency of the laser. The lowest satisfactory value was already at 50 mJ; the next ones were determined by increasing the parameter by a constant 50 mJ.

**Figure 3:**
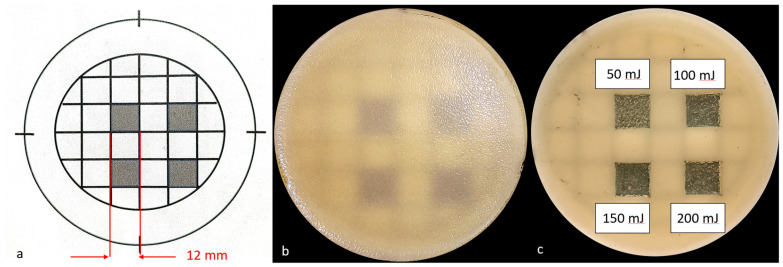
(**a**) Template prepared for the second part of the experiment with 4 visible squares of 12 mm side; (**b**) Petri dish with 96 h *Candida* biofilm standing on template indicating surfaces of Er:YAG laser irradiation (AdverEvo, Morita, Osaka, Japan); (**c**) Petri dish immediately after Er:YAG laser irradiation. The surfaces of individual squares were irradiated with the following energies: upper left—50 mJ, upper right—100 mJ, bottom left—150 mJ, and bottom right—200 mJ.

The irradiation of individual squares began with the preparation of a frame limiting the target area. The time needed to prepare this frame was not included in the designated field development time. The 180 s irradiation of each square area was performed in the following manner, as shown in the diagram shown in Figure 3.

**Figure 4:**
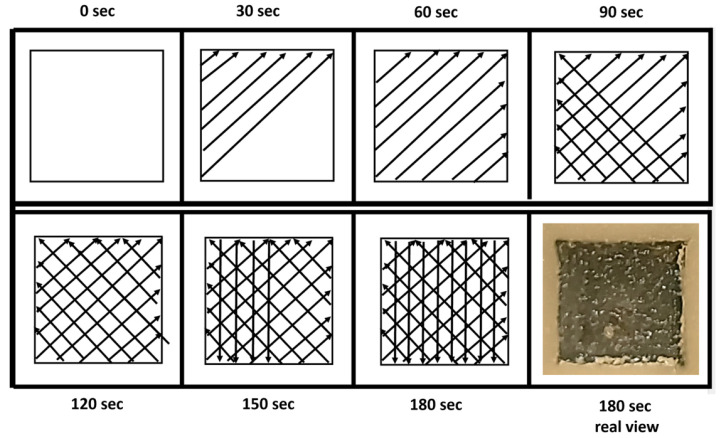
Diagram showing the method of 180 s irradiation of individual squares.

The surface of the laser tip was cleaned every 30 s. The cleaning time was not included in the assumed development. The surfaces of individual squares were processed with the following energies: upper left—50 mJ, upper right—100 mJ, bottom left—150 mJ, and bottom right–200 mJ (Figure 2). Immediately after irradiating all 4 squares, an impression was taken using Rodac IRR LAB-Agar (BioMaxima S.A., Lublin, Poland) (Figure 4). Rodac IRR LAB-Agar is a Sabouraud dextrose agar with chloramphenicol used to determine the total number of yeasts. The plate had a diameter of 55 mm and was gently pressed for 10 s.

After this time, the plate was incubated at 37 °C for 24 h. After incubation, photographic images of the plates were acquired using a D70s Nikon camera from Nikon Corporation (Tokyo, Japan), equipped with a Nikon DX AF-S NIKKOR, 18–70 lens from Nikon Corporation (Tokyo, Japan). These images were taken under white light illumination and using fixed camera settings from a standardized distance of 34 cm.

### 2.8. Statistical Analysis

All quantitative experiments were performed in triplicate or quadruplicate as specified, and, prior to formal statistical testing, data were inspected for normality using the Shapiro–Wilk test (*p* > 0.05 indicating no significant deviation) and for homogeneity of variances using Levene’s test (*p* > 0.05 indicating equal variances), ensuring suitability for parametric tests. Subsequently, one-way ANOVA was conducted to analyse differences in growth inhibition zone measurements across laser energy levels within each *Candida* strain, followed by Newman–Keuls or Tukey’s Honest Significant Difference test for post hoc comparisons if ANOVA was significant, while for colony-forming unit counts in mature biofilms, one-way ANOVA and the same post hoc procedure were applied across four laser energy settings per strain, with all results expressed as mean ± standard deviation (SD), statistical significance set at *p* ≤ 0.05, analyses conducted using Statistica v.7.1 PL (StatSoft Poland, Krakow, Poland), and any outliers identified through a robust outlier test being assessed for experimental error and retained if deemed genuine.

## 3. Results

### 3.1. Evaluation of the Efficacy of the Er:YAG Laser in Inhibiting the Growth of Single-Species Candida Biofilms

In this part of the experiment, growth inhibitory zones (GIZs) were observed on all Petri dishes, regardless of the *Candida* strain tested, at all 32 points irradiated with the Er:YAG laser. The GIZs were already visible at the first scheduled observation time, 48 h after incubation, following laser light exposure, and persisted throughout the entire observation period up to the target of 144 h.

All areas on the Petri dish not irradiated by the laser showed uniform, confluent growth of the unicellular *Candida* biofilm, regardless of the strain tested, throughout the planned observation period. These areas served as the control for this part of the experiment.

**Figure 5:**
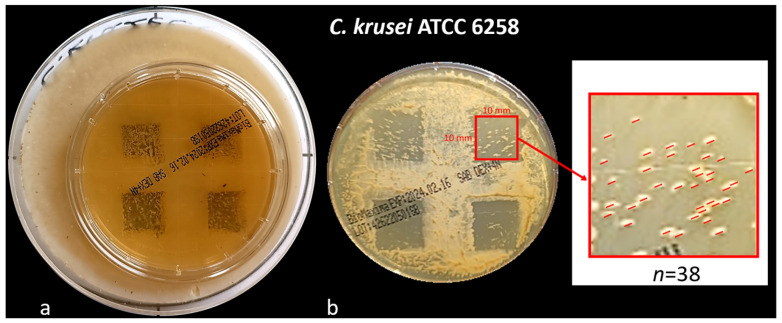
(**a**) Rodac IRR LAB-Agar (Sabouraud dextrose agar with chloramphenicol) gently pressed for 10 s onto the surface of Petri dish exposed to Er:YAG laser; (**b**) Rodac IRR LAB-Agar with *Candida* biofilm after 24 h of incubation at 37 °C. The red frame, 10 × 10 mm, is a way of limiting the area of CFU counting within individual squares. Only those colonies not connected to the square’s edge were considered.

In some observation cases (particularly for the strains *C. parapsilosis* ATCC 22019 and *C. krusei* ATCC 6258), single colony-forming units (CFU) were observed, unrelated to the edge outline of the growth inhibition zone, as early as 48 h, with high laser energy levels (350, 380 mJ). However, such CFUs were also noted with the application of lower energies, e.g., 100 mJ (Figure 5).

**Figure 6:**
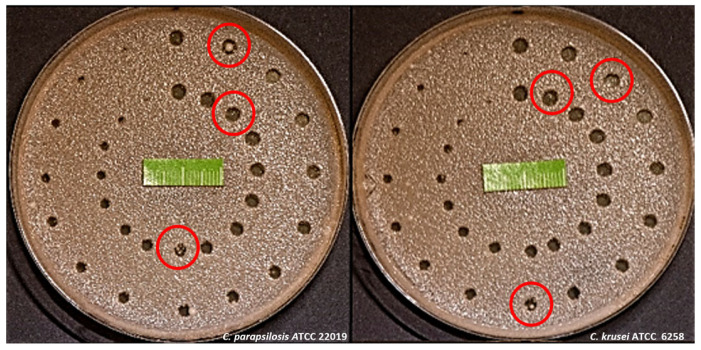
Peri dishes (*C. parapsilosis* ATCC 22019 and *C. krusei* ATCC 6258) with exemplary marked growth inhibition areas (red circles) in which single colony-forming units were observed.

The number of CFUs within the GIZs did not increase over the observation period. Yeast colonies only increased in diameter as the observation time progressed. The width of the GIZ on a single Petri dish varied depending on the laser energy applied and gradually increased from 30 to 400 mJ (Figure 6).

**Figure 7:**
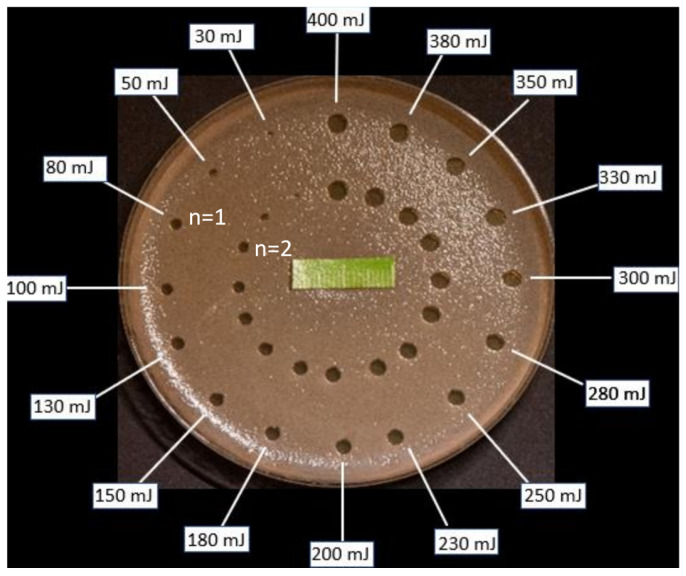
An example Petri dish (*C. krusei* ATCC 6258) illustrates the width of growth inhibition zones (*n* = 1 and *n* = 2, two replicates) for the various energies (30–400 mJ) used in the experiment.

Statistical analysis, expressed as the mean and standard deviation from four repetitions, revealed statistically significant differences in the width of the growth inhibition zones for the different *Candida* strains when the same laser energy was applied (Figure 7).

Statistically, the narrowest GIZ was observed for *C. albicans* ATCC 10231, followed by *C. glabrata* ATCC 90030, *C. parapsilosis* ATCC 22019, and *C. krusei* ATCC 6258. The lowest recorded value was a mean of 1.81 mm for the *C. albicans* ATCC 10231 strain irradiated with 30 mJ energy, while the highest recorded GIZ width was 16.0 mm for the *C. krusei* ATCC 6258 strain irradiated with 400 mJ energy. The smallest and largest results differed significantly, with statistical significance (*p* < 0.0001). Further analyses of the results revealed a rapid increase (statistically significant *p* < 0.001) in the case of increasing the irradiation energy within the range of 30–130 mJ (especially in *C. glabrata* ATCC 90030, *C. parapsilosis* ATCC 22019, and *C. krusei* ATCC 6258 strains). In the energy range of 130–250 mJ, all tested *Candida* strains observed a further statistically significant increase in the width of the growth inhibition zone. The level of statistical significance was *p* < 0.001 for *C. glabrata* ATCC 90030, *C. parapsilosis* ATCC 22019, and *C. krusei* ATCC 6258. A further rise in the energy within the range of 250 to 400 mJ (the maximum for the Morita AdverEvo laser) did not result in a statistically significant increase in the width of the inhibition zone (*p* > 0.05) for *C. albicans* ATCC 10231 and was reduced to *p* < 0.05 for *C. glabrata* ATCC 90030 and *C. parapsilosis* ATCC 22019.

### 3.2. Evaluation of the Efficacy of the Er:YAG Laser in Eliminating Mature Single-Species Candida Biofilms

The variable in this part of the study was the four different energy levels of the laser used. The applied values of 50, 100, 150, and 200 mJ were determined. Values greater than 200 mJ were rejected because they significantly damaged the SDA substrate on which the biofilm was grown (the limit was determined based on a pilot study).

**Figure 8:**
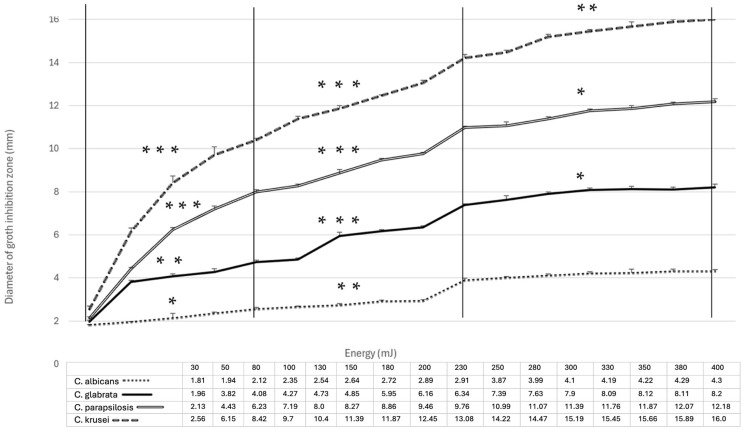
The efficacy of Er:YAG laser in inhibiting the growth of varied single-species *Candida* biofilms, expressed by the diameter of the growth inhibition zone obtained by irradiation of planktonic solution onto Sabouraud dextrose agar plate, with energies in the range of 30–400 mJ. (*) statistical significance level *p* < 0.05; (**) *p* < 0.01; (***) *p* < 0.001.

**Figure 9:**
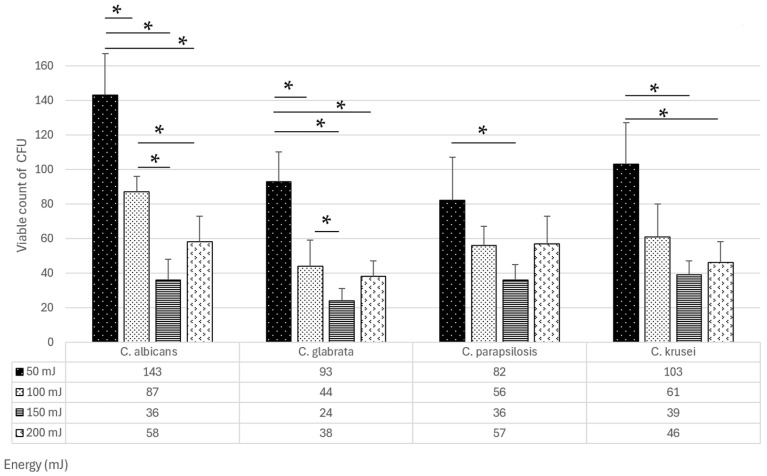
The efficacy of Er:YAG laser in eliminating mature single-species *Candida* biofilms, expressed by the viable count of colony-forming units obtained by irradiation with energy values of 50, 100, 150, and 200 mJ; (*) statistical significance level *p* < 0.05.

After 96 h of prior incubation, the mature *Candida* biofilms showed homogeneous, confluent growth on the surface of the SDA (Figure 2). Subsequently, the number of CFUs that survived laser irradiation within individual squares increased. The sides of each square were reduced from 12 mm to 10 mm due to their uneven edges after incubation. Only those colonies not connected to the square’s edge were considered (Figure 4). Statistical analysis, expressed as the mean and standard deviation from three repetitions (*n* = 3), showed statistically significant differences in the number of surviving CFUs of individual *Candida* strains when the same laser energy was applied (Figure 8 and Figure 9).

Statistically, the highest number of surviving CFUs was observed for *C. albicans* ATCC 10231 after irradiation with 50 mJ (143 ± 24). In contrast, the lowest number was recorded for the *C. glabrata* ATCC 90030 strain at 150 mJ energy (24 ± 7). The smallest and largest results differed significantly with statistical significance (*p* < 0.0005). Further analyses showed that the fewest CFUs survived during biofilm irradiation with 150 mJ energy, regardless of the observed *Candida* strain. The results for 150 mJ energy did not differ significantly (*p* > 0.05) between the tested *Candida* strains; however, they differed significantly (*p* < 0.05) from 50 mJ energy (all tested *Candida* strains) and 100 mJ energy (*C. glabrata* ATCC 90030).

## 4. Discussion

### 4.1. The Significance of the Results

The present study investigated the use of an Er:YAG laser at various energy settings for the inhibition and elimination of single-species *Candida* biofilms. The key outcomes revealed that Er:YAG laser irradiation could inhibit biofilm growth even at a relatively low energy (50 mJ) and significantly reduce mature biofilms most effectively at 150 mJ. These findings support the initial hypothesis that the Er:YAG laser exhibits promising antifungal activity against *Candida* spp. The results thus reject the null hypothesis that there is no difference in disinfection efficacy at different laser energies. When interpreted in light of the current literature, these outcomes underscore the laser’s capacity to disrupt fungal structures primarily due to its high absorption in water-containing tissues. Previous research demonstrated that the Er:YAG laser effectively damages fungal cell walls and cytoplasmic contents even without direct contact, as reported by Sennhenn-Kirchner et al. [62]. Our results align with these findings, indicating that Er:YAG laser irradiation could be a valuable approach for local disinfection in oral cavities colonized by *Candida*, especially where conventional antifungal therapies have failed or adjunctive measures are necessary. Despite these encouraging results, the present study did not directly compare the Er:YAG laser to conventional antifungal regimens or emerging photodynamic therapy (PDT). This lack of comparison, while beyond the initial scope, is a limitation that may affect how clearly the advantages of the Er:YAG laser are highlighted. Traditional antifungal drugs, such as azoles or polyenes, are widely used but face increasing challenges due to resistance and recurrence. Photodynamic therapy, on the other hand, has shown the potential for reducing *Candida* biofilm viability through reactive oxygen species generated in the presence of photosensitizers. While both pharmacological agents and aPDT can be effective, they differ from Er:YAG laser therapy in their dependence on chemical agents, potential systemic side effects, or local damage [69,70]. In contrast, Er:YAG laser disinfection relies on physical ablation and photothermal effects, potentially offering a faster, localized solution without introducing exogenous photosensitizers. Future studies designed as head-to-head comparisons of the Er:YAG with antifungal drugs and photodynamic therapy would elucidate the distinct advantages, potential synergies, and best clinical applications for each modality [63,64,65,66,67,68]. In clinical settings, Candida species rarely exist in isolation; they often form multi-species biofilms with bacteria such as *Streptococcus gordonii*, *S. salivarius*, and *S. oralis* [59]. These biofilms exhibit increased resistance to antifungal treatments and can complicate oral infections [64]. While this study focused on single-species biofilms, the efficacy of the Er:YAG laser in disrupting mixed fungal–bacterial biofilms remains uncertain. Future investigations should explore whether Er:YAG laser treatment can effectively penetrate and eradicate these more complex biofilms. Additionally, understanding the potential synergistic or antagonistic interactions between Candida and oral bacteria during laser therapy would be essential for optimizing its clinical use [64,65,66,67,68,69].

Notably, while the Er:YAG laser showed high antifungal efficacy, it also caused damage to the agar substrate at higher doses (≥200 mJ). This raises the question of whether similar tissue damage could occur within oral tissues, particularly the oral mucosa. Although our in vitro model could not directly evaluate host cell safety, thermal damage to oral tissues is a valid concern. Some clinical studies on Er:YAG lasers in soft tissue surgery have demonstrated minimal thermal side effects due to the high absorption of the 2940 nm wavelength in water and the use of water cooling [65,66,67]. However, our study did not measure temperature rises or histological changes in host tissues. Investigations assessing the extent of thermal damage in vivo—perhaps through ex vivo porcine mucosa models or real-time temperature measurements—would address this safety concern and provide a more comprehensive safety profile for long-term use. A further consideration is the role of multi-species biofilms in clinical candida infections. Oral biofilms frequently comprise both fungal and bacterial species, forming consortia more resistant to treatment than single-species biofilms [40,71,72,73]. While our data demonstrate the capacity of the Er:YAG laser to effectively disrupt *Candida* in a single-species setting, its impact on mixed biofilms—including possible synergy with or antagonism toward certain bacterial pathogens—remains uncertain. Studies incorporating complex in vitro biofilm models or in vivo clinical trials could yield a more realistic view of how Er:YAG laser application translates into patient outcomes. While our in vitro results demonstrate the antifungal efficacy of Er:YAG laser therapy, translating these findings into clinical practice requires further validation [44]. The potential use of the Er:YAG laser as an adjunctive or alternative therapy to antifungal drugs in conditions such as denture stomatitis, oropharyngeal candidiasis, and peri-implantitis could be beneficial for patients with recurrent or drug-resistant infections [67,68,69]. However, future clinical trials are necessary to assess treatment efficacy, safety, and patient outcomes in real-world settings. Additionally, the standardization of laser parameters for clinical application, including energy settings, irradiation time, and frequency, is crucial to ensure optimal therapeutic results [69,70,71,72,73].

### 4.2. Limitations and Future Directions

This study was performed under laboratory conditions using single-species biofilms, which do not fully replicate the complexities of the oral cavity, necessitating future investigations in multi-species biofilms and clinical settings. Additionally, while our findings showed that 200 mJ caused damage to the agar substrate, no direct assessment of potential thermal or mechanical injury to oral tissues was conducted, highlighting the need for temperature monitoring and histological evaluations to establish safety thresholds.

The damaging effect on tissues was proven by other studies such as that by Altschuler et al., which demonstrated that the 200 mJ Er:YAG laser can be effectively used for enamel removal, with the pneumo-laser method improving efficiency by 3.2 times compared to traditional techniques by alternating water and air blasts to prevent debris buildup and enhance cutting performance [74].

The study also focused solely on the efficacy of the Er:YAG laser without direct comparison to standard antifungal agents, such as nystatin or fluconazole, or emerging treatments like photodynamic therapy, making such comparisons crucial for contextualizing its therapeutic role. Furthermore, the long-term stability of laser-induced biofilm reductions remains unassessed, and since candida infections frequently recur, extended observation periods are necessary to determine the durability of clinical benefits. Although 150 mJ was the most efficacious setting in our experiments, the further optimization of laser parameters—such as energy, frequency, pulse duration, and irradiation time—is needed to balance tissue safety with antifungal effectiveness, potentially through clinical trials exploring variable parameters. Given these limitations, future research should focus on developing robust in vivo studies that compare Er:YAG laser therapy directly with pharmacological agents and photodynamic therapy while investigating its interactions with complex, multi-species oral biofilms, evaluating safety margins for oral tissues and assessing the durability of antifungal effects over extended periods. Such research would build upon the promising in vitro data presented here, offering a comprehensive picture of the Er:YAG laser’s potential role as a novel or adjunctive treatment modality for oral candidiasis.

This study demonstrates that Er:YAG laser irradiation at low- to moderate-energy levels can inhibit the growth of various *Candida* strains and substantially reduce mature biofilms. These results uphold our study hypothesis, showing that a 150 mJ setting may provide an optimal balance between disinfection efficacy and potential substrate damage in vitro. Despite the encouraging findings, additional studies—particularly those comparing the Er:YAG laser with conventional and emerging antifungal approaches and addressing host safety—are essential to confirm the clinical viability and long-term benefits of this method.

A critical limitation of this study is the absence of direct comparison between Er:YAG laser therapy and conventional antifungal treatments. Azoles such as fluconazole and polyenes like amphotericin B remain the standard of care for Candida infections, but increasing resistance has necessitated the exploration of alternative strategies. While photodynamic therapy (PDT) and novel antifungal agents have been investigated, Er:YAG laser therapy presents a unique non-pharmacological approach that may be particularly useful for drug-resistant Candida strains. Comparative studies evaluating fungal reduction rates, recurrence rates, and tissue healing between Er:YAG laser and standard antifungal regimens would provide a more comprehensive understanding of its clinical value.

## 5. Conclusions

Our results clearly reject this null hypothesis. Even at relatively low-energy settings (50 mJ/0.5 W), the Er:YAG laser significantly inhibited biofilm growth, and at a specific energy level of 150 mJ, it achieved the most effective reduction in mature biofilms (*C. albicans*, *C. glabrata*, *C. parapsilosis*, and *C. krusei*). By demonstrating these antifungal properties across diverse *Candida* strains, the Er:YAG laser appears to be a promising adjunctive or alternative strategy, especially where standard antifungal therapies are ineffective or contraindicated. However, the translation of these findings from single-species, in vitro biofilms to clinical practice remains contingent on further in vivo studies and head-to-head comparisons with pharmacological antifungal regimens. Future investigations should also address the complexity of multi-species biofilms and diverse patient backgrounds to optimize the clinical parameters for laser use in managing candidiasis. Despite demonstrating significant antifungal efficacy, the long-term stability of Er:YAG laser-induced biofilm reductions and its clinical safety profile warrant further investigation. Future research should focus on in vivo models to assess potential adverse effects on oral tissues, particularly thermal damage risks. Additionally, evaluating the Er:YAG laser’s effectiveness in multi-species biofilms and comparing it with standard antifungal therapies would enhance its translational potential. If confirmed in clinical trials, Er:YAG laser therapy could serve as an adjunct or alternative strategy, particularly for patients with recurrent or drug-resistant oral Candida infections.

## Data Availability

Data are contained within the article.
